# Anti-DFS70 Antibodies Among Patient and Healthy Population Cohorts in China: Results From a Multicenter Training Program Showing Spontaneous Abortion and Pediatric Systemic Autoimmune Rheumatic Diseases Are Common in Anti-DFS70 Positive Patients

**DOI:** 10.3389/fimmu.2020.562138

**Published:** 2020-10-02

**Authors:** Bing Zheng, Zhiqing Wang, Rodrigo A. Mora, Aiping Liu, Chihui Li, Dengtao Liu, Fuying Zhai, Huiyuan Liu, Huiyun Gong, Jiaye Zhou, Jing Liu, Li Chen, Lijun Wu, Lin Yuan, Lina Ying, Loujian Jie, Meifang He, Meng Hao, Ping Xu, Qiuwei Lu, Shanshan Han, Shishi Chen, Shuimian Chen, Shunfei Zhu, Weihua Sun, Xiaoying Guo, Yajuan Chen, Yan Wang, Yemin Qu, Zhen Li, Zhenzhen Niu, Zhongyan Han, Edward K. L. Chan

**Affiliations:** ^1^Department of Laboratory Medicine, Renji Hospital, School of Medicine, Shanghai Jiao Tong University, Shanghai, China; ^2^Department of Oral Biology, University of Florida, Gainesville, FL, United States; ^3^Department of Laboratory Medicine, Huashan Hospital, Fudan University, Shanghai, China; ^4^Department of Laboratory Medicine, Jinhua People’s Hospital, Jinhua, China; ^5^Department of Clinical Laboratory, Linyi People’s Hospital, Linyi, China; ^6^Department of Laboratory Medicine, People’s Hospital of Rongcheng, Rongcheng, China; ^7^Department of Rheumatology Laboratory, Futian District Hospital of Rheumatism, Shenzhen, China; ^8^Department of Laboratory Medicine, Ruijing Hospital, School of Medicine, Shanghai Jiao Tong University, Shanghai, China; ^9^Department of Laboratory Medicine, Zhongshan Hospital, Fudan University, Shanghai, China; ^10^Department of Laboratory Medicine, General Hospital of Northern Theater Command, Shengyang, China; ^11^Department of Laboratory Medicine, Zhejiang Provincial Hospital of TCM, The First Affiliated Hospital of Zhejiang Chinese Medicine University, Hangzhou, China; ^12^Department of Rheumatology Laboratory, People’s Hospital of Xinjiang Ugyur Autonomous Region, Urumqi, China; ^13^Department of Laboratory Medicine, Weihai Central Hospital, Weihai, China; ^14^Department of Clinical Laboratory, Ninbo No.6 Hospital, Ninbo, China; ^15^Department of Laboratory Medicine, Affiliated Dongyang Hospital of Wenzhou Medical University, Dongyang, China; ^16^Department of Laboratory Medicine, The Third People’s Hospital of Changzhou, Changzhou, China; ^17^Department of Laboratory Medicine, Jiujiang First People’s Hospital, Jiujiang, China; ^18^Department of Laboratory Medicine, Obstetrics & Gynecology Hospital Affiliated to Fudan University, Shanghai, China; ^19^Department of Laboratory Medicine, The People’s Hospital of Guangxi Zhuang Autonomous Regain, Nanning, China; ^20^Department of Laboratory Medicine, People’s Hospital of Sanmen, Taizhou, China; ^21^Department of Laboratory Medicine, The Fifth Affiliated Hospital Sun Yat-Sen University, Zhuhai, China; ^22^Department of Laboratory Medicine, Zhenggu Hospital, School of Medicine, Fujian University of Traditional Chinese Medicine, Quanzhou, China; ^23^Department of Laboratory Medicine, Zibo Maternal and Child Health Hospital, Zibo, China; ^24^Department of Laboratory Medicine, The 940th Hospital of PLA Joint Logistics Support Force, Lanzhou, China; ^25^Department of Laboratory Medicine, Daqing Oilfield General Hospital, Daqing, China; ^26^Departments of Microbiological and Immunology, 3201 Hospital, Hanzhong, China; ^27^Department of Laboratory Medicine, The Red Cross Hospital, Xining, China; ^28^Department of Laboratory Medicine, Weihai Municipal Hospital, Shandong University, Weihai, China; ^29^Department of Laboratory Medicine, Yili Kazak Autonomous Prefecture Hospital of Traditional Chinese Medicine, Yili, China; ^30^Department of Laboratory Medicine, Yinzhou People’s Hospital, Ningbo, China; ^31^Center of Pathology and Clinical Laboratory, Sir Run Run Hospital, Nanjing Medical University, Nanjing, China

**Keywords:** antinuclear antibodies, DFS70, pediatric rheumatic disease, spontaneous abortion, systemic autoimmune rheumatic disease

## Abstract

**Objective:**

Anti-DFS70 antibodies correlating with the nuclear dense fine speckled (DFS) pattern in the HEp-2 indirect immunofluorescence assay (IFA) are less common in patients with systemic autoimmune rheumatic disease (SARD) than in healthy subjects and their clinical associations remain elusive. We hosted a multi-center HEp-2 IFA training program to improve the ability of clinical laboratories to recognize the DFS pattern and to investigate the prevalence and relevance of anti-DFS70 antibodies.

**Methods:**

DFS pattern sera identified by HEp-2 IFA in 29 centers in China were redirected to a central laboratory for anti-DFS70 testing by line immunoblot assay (LIA), enzyme-linked immunosorbent assay (ELISA), and IFA with HEp-2 ELITE/DFS70-KO substrate. Anti-extractable nuclear antigen antibodies were measured by LIA and the clinical relevance was examined in adult and pediatric patients.

**Results:**

HEp-2 IFA positive rate and DFS pattern in positive sera were 36.2% (34,417/95,131) and 1.7% (582/34,417) in the patient cohort, and 10.0% (423/4,234) and 7.8% (33/423) in a healthy population, respectively. Anti-DFS70 prevalence among sera presenting the DFS pattern was 96.0, 93.7, and 49.6% by ELISA, LIA, and HEp-2 ELITE, respectively. 15.5% (52/336) of adult and 50.0% (20/40) of pediatric anti-DFS70 positive patients were diagnosed with SARD. Diseases most common in anti-DFS70 positive patients were spontaneous abortion (28.0%) in adults and juvenile idiopathic arthritis (22.5%) in pediatric patients.

**Conclusion:**

Accurate DFS pattern identification increased the detection rate of anti-DFS70 antibodies by ELISA and LIA. Anti-DFS70 antibodies are remarkably high in cases of spontaneous abortion and in pediatric SARD patients, but not prevalent in adult SARD patients.

## Introduction

Antinuclear antibodies (ANA) are commonly regarded as serological hallmarks of systemic autoimmune rheumatic disease (SARD) (1). However, antibodies against the 70 kDa dense fine speckled protein (DFS70), also known as transcription coactivator p75 (2) and lens epithelium-derived growth factor (3), are purported to be an interesting immunological paradox as they are commonly detected in apparently healthy individuals (4–9) but are rare in patients with SARD (5, 10). In the last decade, many studies have focused on the clinical relevance of anti-DFS70 antibodies [reviewed in (5)] and their prevalence has been reported in some chronic inflammatory diseases (2, 11, 12) and cancers [e.g. prostate cancer (13, 14)], but still no clear disease association has been found. In fact, the presence of isolated anti-DFS70 antibodies has been proposed to serve as a diagnostic biomarker to help rule out SARD (4, 15, 16), which highlights the importance of correctly identifying these antibodies in clinical laboratories.

The HEp-2 cell indirect immunofluorescence assay (HEp-2 IFA) is considered the “gold standard” method for ANA screening by the American College of Rheumatology (17). Typical images of the DFS pattern show dense fine speckled staining of interphase nuclei and strong coarse speckled staining of the metaphase plate. DFS pattern is defined as the anti-cell-2 (AC-2) pattern by the International Consensus on ANA Patterns (ICAP) (18). Due to its unique features, only trained and experienced technicians may recognize DFS pattern. Correlations between the anti-DFS70 antibodies detected by specific assays and the DFS pattern have been reported higher than 90% (19). Considering HEp-2 IFA pattern interpretation is largely dependent on the experience of the technologist, reading of the DFS pattern continues to challenge researchers and clinicians alike. Bentow et al. reported that the reading accuracy of DFS unmixed and mixed patterns were ~50% and <10%, respectively, based on an international internet-based survey (20). Recently, the Autoantibody Standardization Committee has made available a reference material for anti-DFS70 antibodies (21), which may assist clinical laboratories in the recognition of DFS pattern to some extent. However, further efforts still need to be placed on training to recognize the DFS pattern.

Our research focused on exploring cost-effective training models to improve the consistency of DFS pattern recognition in laboratories from various regions with unevenly distributed medical resources. Thus, we organized a multi-center DFS pattern identification mutual aid program. The prevalence and clinical associations of anti-DFS70 in both Han Chinese and minority populations were investigated in routine HEp-2 IFA screening cohorts from 30 centers and in healthy individuals from a physical examination cohort from one center in China.

## Materials and Methods

### Study Design

**Figure 1:**
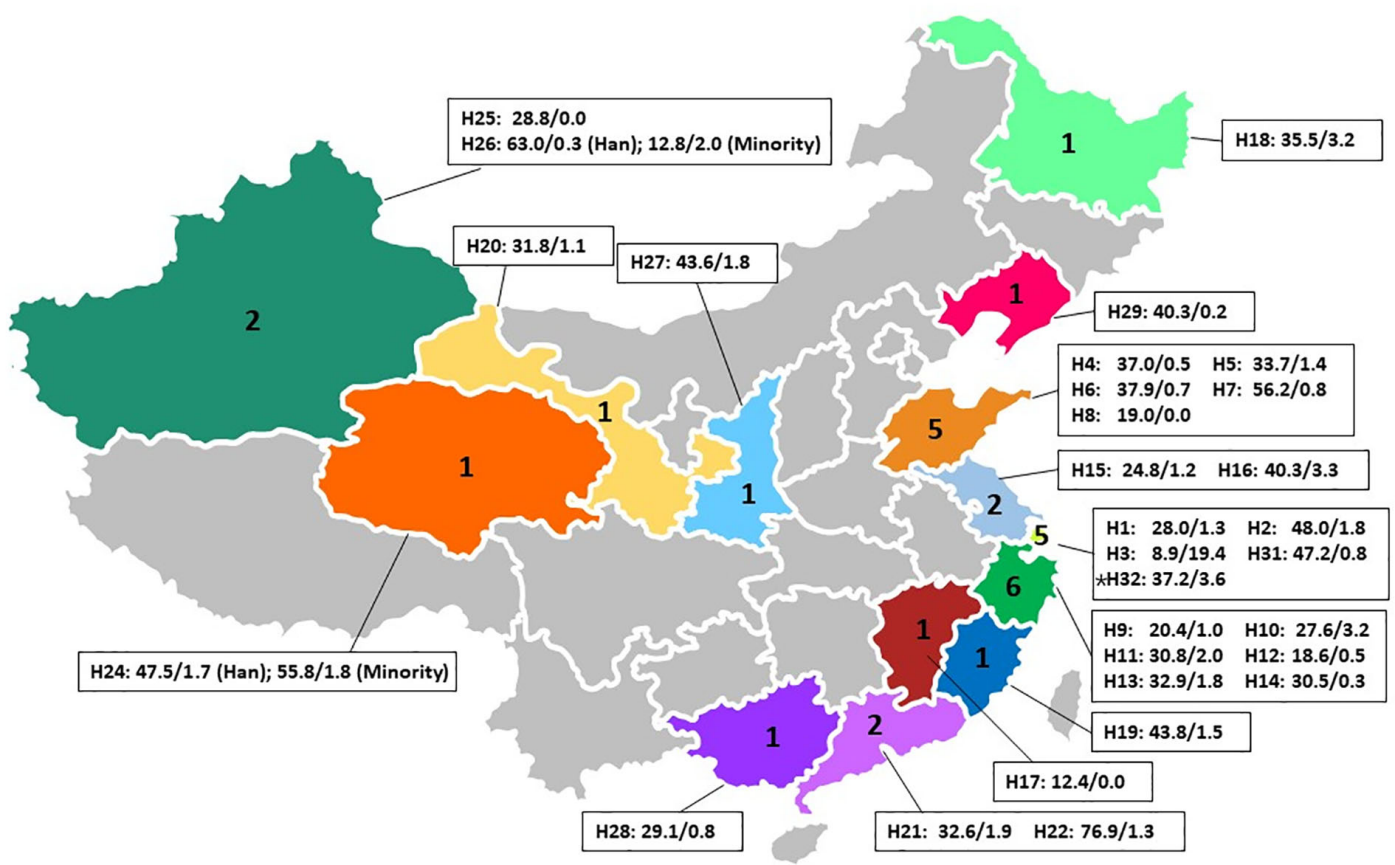
Distribution of one central laboratory ‘(*H32)’ and 29 participating laboratories across China and the positive rates of the HEp-2 IFA screening test and DFS pattern in IFA positive sera. H(number) is the ID for diﬀerent centers as listed in ‘**Table 1**’. Following the code name is the HEp-2 IFA positive rate (%)/DFS pattern rate in IFA positive sera (%). Han: Han Chinese. Minorities in H24 included Tibetans, Huis, and Mongolians. Minorities in H26 included Uygurs and Kazakhs.

From July to September 2019, a total of 645 serum specimens were sent from 29 research centers across China to the organizing laboratory at the Renji Hospital, which is affiliated with Jiao Tong University (Shanghai, China). **Figure 1** and **Table 1** detail the distribution and specific information (e.g. type of hospital, location, etc.) of the 30 participating laboratories.

**Table 1:** Detailed information of HEp-2 IFA positive and DFS pattern rates of one central laboratory and 29 participating centers during a 3-month study period.

ID	Type	Location	Routinely reported DFS pattern	HEp-2 IFA assayed (n)	Positiven (%)	DFS pattern		
						Reported (n)^a^	Confirmed (n)^b^	Rate (%)^c^
H1	General	Shanghai	N	5440	1525 (28.0)	19	19£	1.3
H2	General	Shanghai	N	1597	766 (48.0)	21	14	1.8
H3	OB/GYN	Shanghai	N	1051	93 (8.9)	23	18	19.4
H4	General	SD	N	541	200 (37.0)	4	1	0.5
H5	General	SD	N	3715	1253 (33.7)	19	17	1.4
H6	General	SD	N	715	271 (37.9)	5	2	0.7
H7	General	SD	N	1124	632 (56.2)	31	5	0.8
H8	OB/GYN	SD	N	100	19 (19.0)	0	0	0.0
H9	TCM	ZJ	N	3784	770 (20.4)	31	8£	1.0
H10	General	ZJ	N	1362	376 (27.6)	31	12	3.2
H11	General	ZJ	N	331	102 (30.8)	13	2	2.0
H12	General	ZJ	N	2282	425 (18.6)	19	2	0.5
H13	General	ZJ	N	2575	846 (32.9)	28	15	1.8
H14	General	ZJ	N	6690	2039 (30.5)	8	5	0.3
H15	General	JS	N	2791	691 (24.8)	22	8	1.2
H16	General	JS	N	305	123 (40.3)	7	4	3.3
H17	General	JX	N	1867	231 (12.4)	15	0£	0.0†
H18	General	HLJ	N	1515	538 (35.5)	85	17	3.2
H19	TCM	FJ	N	468	205 (43.8)	17	3	1.5
H20	General	GS	N	1441	458 (31.8)	20	5	1.1
H21	General	GD	N	2909	948 (32.6)	19	18	1.9
H22	RSH	GD	N	2276	1750 (76.9)	41	23	1.3
H24	General	QH	N	861 (Han)	409 (47.5)	16	7	1.7
				582 (MP)	325(55.8)	15	6	1.8
H25	TCM	XJ	N	593	171 (28.8)	1	0	0.0
H26	General	XJ	Y	3697(Han)	2328 (63.0)	19	8	0.3
				2719 (MP)	347(12.8)	14	7	2.0
H27	General	SX	N	917	400 (43.6)	9	7	1.8
H28	General	GX	N	5195	1512 (29.1)	32	12£	0.8^†^
H29	General	LN	N	1244	501 (40.3)	2	1	0.2
H31	General	Shanghai	N	13395	6325 (47.2)	59	52	0.8
H32*	General	Shanghai	N	21049	7838 (37.2)	284	284	3.6
Total				95131	34,417 (36.2)	930	582	1.7

OB/GYN, Obstetrics and Gynecology Hospital; RSH, Rheumatology specialist hospital; TCM, Traditional Chinese Medicine hospital; SD, Shandong Province; ZJ, Zhejiang Province; JS, Jiangsu Province; JX, Jiangxi Province; HLJ, Heilongjiang Province; FU, Fujian Province; GS, Gansu Province; GD, Guangdong Province; QH, Qinghai Province; XJ, Xinjiang Province; SX, Shanxi Province; GX, Guangxi Province; LN, Liaoning Province; Han, Han Chinese; MP, Minority populations.

MP in H24 included Tibetans, Huis, and Mongolians.

MP in H26 included Uygurs and Kazakhs.

^a^DFS pattern serum reported by participating centers; ^b^DFS pattern confirmed by Renji Hospital after retesting the samples received from the participating centers using the same commercial slides (￡: DFS pattern confirmed by Inova, the rest were confirmed by Euroimmun); ^c^Positive rate of DFS pattern in ANA positive samples in each center. The DFS pattern cases were calculated by the samples confirmed by Renji Hospital.

^†^DFS pattern confirmed by Renji Hospital using Inova slides, which was different from that used it the participating hospital.

*H32 is the central laboratory in this research.

**Figure 2:**
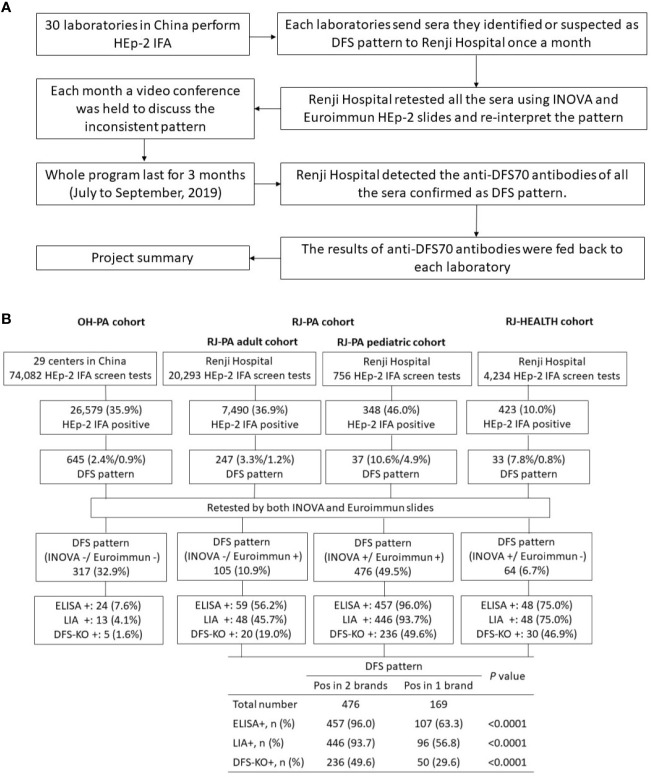
Summary of DFS pattern recognition and training project. **(A)** Project workflow for organizing sample referrals sent to Renji Hospital from participating training laboratories for validation of DFS pattern and detection of anti-DFS70 antibody. **(B)** Summary of results of the HEp-2 cell indirect immunofluorescence assay (HEp-2 IFA) and anti-DFS70 antibody assays in three Chinese cohorts collected between July and September 2019. The patient cohort from other hospitals (OH-PA) included samples from 29 participating laboratories in China, excluding the organizing laboratory at Renji Hospital. The RJ-PA cohort included both adult and pediatric patients subjected to routine HEp-2 IFA screening in Renji Hospital. The RJ-HEALTH cohort represents the generally healthy population in the Physical Examination Center of Renji Hospital during the same period of this project. DFS pattern positive rates were shown by the percentage of DFS pattern in HEp-2 IFA positive sera/DFS pattern in HEp-2 IFA screen tests. All samples were re-tested by two commercial HEp-2 IFA kits and further analyzed by ELISA, LIA, and IFA on ELITE/DFS KO substrate. The positive rates of anti-DFS70 were compared between sera showing DFS pattern in both or either one of the commercial HEp-2 IFA kits. Pos in 2 brands: DFS pattern was observed in both Inova and Euroimmun slides. Pos in 1 brand: DFS patterns were observed only in Inova or Euroimmun. ELISA: enzyme-linked immunosorbent assay; LIA: line immunoblot assay; DFS-KO: IFA performed on HEp-2 ELITE/DFS70-KO substrate.

At the initiation of the study, corresponding author BZ at the Renji Hospital organizing center directed an online training course to all participating laboratories detailing how to identify the DFS pattern based on the ICAP classification criteria (22). In brief, interphase nuclei show characteristic speckles with heterogeneity in brightness and distribution, while the metaphase plate depicts a strong speckled pattern with some coarse speckles (22). At the end of each month, participating laboratories shipped sera they interpreted as DFS pattern positive during routine HEp-2 IFA tests to Renji Hospital. Upon arrival, two experienced research technicians re-tested the samples by HEp-2 IFA and reported patterns according to ICAP classification (22). Inconsistent interpretations were discussed between two independent evaluators. If they could not reach a consensus, a third evaluator adjudicated. A video conference was offered monthly to review all inconsistent ANA pattern interpretations as part of the ongoing training. **Figure 2A** shows a flowchart of the study design.

Additionally, the prevalence of ANA and DFS patterns were analyzed in a healthy population (age≥18) from the Renji Hospital Physical Examination Center for routine physical examination (RJ-HEALTH cohort, n=4,234) from July to September 2019. This cohort according to a clinical questionnaire had no history of SARD, malignancies or chronic infections, and were considered healthy by judgment of the chief physician based on physical examination reports. This healthy cohort was compared to the adults in RJ-PA cohort, which consisted of adult (≥18 years, n=20,293) and pediatric (<18 years, n=756) patients undergoing routine HEp-2 IFA screening in the same hospital during the same period (RJ-PA cohort, n=21,049). The percentage of SARD patients in RJ-PA cohort were also investigated according to medical record. All serum specimens collected in this study were approved by the Institutional Review Board of Renji Hospital (No. KY[2019]121). No consent was required for this study.

### ANA Screening

Of the 30 participating laboratories, including Renji Hospital, 25 of the laboratories performed ANA screening on HEp-2 slides from Euroimmun AG (Lübeck, Germany), 3 used slides from Inova Diagnostics (San Diego, CA, USA), and 2 used HEp-2 IFA kits from two companies in mainland China (H&J NovoMed, Beijing and HOB, Suzhou).

All samples received by the organizing center at Renji Hospital were re-tested using HEp-2 slides from both Euroimmun and Inova Diagnostics. Euroimmun HEp-2 slides were prepared using the Sprinter XL automated IFT/ELISA analyzer (Euroimmun). Images were acquired with EUROPattern Suite version 3.4.24.0 with a cutoff of 80 AU per the manufacturer’s instructions. NOVA Lite HEp-2 IgG ANA with DAPI slides were processed using the IFA-ELISA processor platform of the QUANTA-Lyser instrument (Inova Diagnostics) and scanned *via* the NOVA View automated microscope with software version 1.0.4.3, using a cutoff of 49 nuclear intensities (NI) per the manufacturer’s instructions. Samples positive for any nuclear/cytoplasmic autoantibody by IFA were considered HEp-2 IFA positive. Anti-DFS70 reference material IS2726 ANA #24 (21) for ICAP pattern AC-2 was applied as the quality control for HEp-2 IFA.

### Anti-DFS70 Antibodies Detection

Anti-DFS70 antibodies were detected by both line immunoblot assay (LIA) and enzyme-linked immunosorbent assay (ELISA). For LIA, the IMTEC-ANA-LIA XL assay (Human Worldwide, Weisbaden, Germany) was used. HumaScan was used to analyze and interpret the results according to the manufacturer’s instructions. For ELISA, 96-well plates were coated with 0.5 µg/mL purified recombinant DFS70 antigen (DIARECT AG, Freiburg, Germany) overnight at 4°C. After blocking with 10 mg/mL gelatin, patient sera were diluted 1:200 in serum diluent (0.75 mg/mL bovine gamma globulin, 0.15 mg/mL bovine serum albumin, 10 mg/mL gelatin, 0.05% Tween 20, 0.5M phosphate buffer, pH 7.4) and added to each well for 2 h incubation at room temperature. For the secondary antibody, horseradish peroxidase-conjugated AffiniPure rabbit anti-human IgG (Jackson ImmunoResearch, West Grove, PA) was diluted 1:1,000 in serum diluent. Samples were developed by TMB (3,3′,5,5′-tetramethylbenzidine, Euroimmun) and the optical density (O.D.) value was read at 450 nm by microplate reader (Multiskan FC, Thermo Fisher, Waltham, MA, USA). Sixty serum samples showing negative reaction to DFS70 by IMTEC-ANA-LIA XL assay (Human Worldwide) were used as negative controls. These included twenty ANA negative from healthy population and forty homogenous and speckled patterns from SARD patients with various titers by HEp-2 IFA. Cutoff was determined by mean O.D. value +3 standard deviations (SD). Anti-DFS70 reference material (21) was used as the positive control for both assays.

### Immunofluorescence Assay Using HEp-2 ELITE/DFS70-KO Substrate

IFA was also performed using HEp-2 ELITE/DFS70-KO slides (Immco Diagnostics, Trinity Biotech, Buffalo, NY, USA), which consist of a mixture of 10% conventional HEp-2 cells and 90% engineered HEp-2 cells that do not express DFS70 antigen (23). Results were interpreted using a manual fluorescence microscope (Nikon Eclipse Ni, Tokyo, Japan). Anti-DFS70 antibodies were confirmed by bright staining of interphase nuclei with the corresponding DFS pattern in ~10% of cells.

### Line Immunoblot Assay (LIA)

To examine potential association with other autoantibodies, sera positive for anti-DFS70 by ELISA and LIA were further screened for fifteen autoantibodies (nRNP/Sm, Sm, SSA/Ro60, Ro52/TRIM21, SSB/La, Scl-70, PM-Scl, Jo-1, CENP-B, PCNA, dsDNA, nucleosomes, histones, ribosomal P protein (Rib-P), AMA-M2) using the Euroline ANA Profile 3 (Euroimmun) according to the manufacturer’s instructions.

### Questionnaire for HEp-2 IFA Screening in Participating Centers

The following were determined by questionnaire for each participating center: which commercial kits they used for routine HEp-2 IFA screening; whether the laboratory routinely reports the DFS pattern; the working experience of their research technicians; whether they apply third-party quality control for HEp-2 IFA. Moreover, the clinical diagnoses of sera with the DFS pattern, which were re-tested and confirmed by Renji Hospital, were retrospectively analyzed by reviewing the medical records including the age, gender, ethnicity and clinical diagnosis from each center.

### Statistical Analysis

Statistical Package for Social Sciences (SPSS) (IBM-SPSS, Inc., Armonk, New York) was used to perform statistical analysis. Cohen’s kappa was applied to analyze the interrater agreement between two commercial slides. Differences in the distribution of anti-extractable nuclear antigen (ENA) between SARD versus non-SARD were evaluated by the two-tailed Chi-squared (χ^2^) test or Fisher’s exact test. The correlations between anti-DFS70 ELISA O.D. values and HEp-2 IFA nuclei fluorescence intensity read by NOVA View or titer by HEp-2 IFA were calculated by Spearman’s rank correlation test. In our study, a two-sided t-test with a *P*-value <0.05 was considered significant.

## Results

### Prevalence of ANA and DFS Pattern in China

The 30 participating laboratories consisted of one rheumatology specialist hospital in southern China, three traditional Chinese medicine hospitals in eastern and western China, two obstetrics and gynecology specialist hospitals in eastern China, and twenty-four general hospitals across mainland China. Each laboratory was assigned a unique hospital identification number for the study as listed in **Table 1**. Overall, the HEp-2 IFA positive rate was 36.2% (34,417/95,131) with some significant differences among the centers. For example, rheumatology specialist hospital H22 had the highest HEp-2 IFA positive rate (76.9%), while obstetrics and gynecology specialist hospital H3 had the lowest rate (8.9%, **Table 1**).

The prevalence of the DFS pattern was 0.6% (582/95,131) of total patients and 1.7% (582/34,417) in the HEp-2 IFA positive samples, respectively. Interestingly, an obstetrics and gynecology specialist hospital (H3) had the lowest overall HEp-2 IFA positive rate (8.9%), but the highest positive rate of DFS pattern (19.4%) among the participating centers. Note that this high percent of positive DFS pattern could not be accounted for by unusual local environmental exposure when compared to four neighboring centers in the same region (H1, H2, H31, and H32, **Figure 1**). Three centers, including another obstetrics and gynecology specialist hospital (H8), a Traditional Chinese Medicine hospital (H25), and a general hospital (H17), did not have any confirmed samples with DFS pattern.

To determine the effects of geographical location and potential differences in dietary and lifestyle choices, HEp-2 IFA results were compared between local Han Chinese and minority populations within the H24 and H26 cohorts (**Table 1**). In H24, the HEp-2 IFA positive rate was higher among the minority population (325/582, 55.8%), which included Tibetans, Huis, and Mongolians than in Han Chinese (409/861, 47.5%, *P*=0.002). However, the prevalence of the DFS pattern in HEp-2 IFA positive samples was similar in both groups (Han: 7/409, 1.7%; minorities: 6/325, 1.8%, *P*=0.891). In H26, the HEp-2 IFA positive rate was significantly lower among the minority population (347/2,719, 12.8%) than in Han Chinese (2,328/3,697, 63.0%, *P*<0.001), yet the prevalence of the DFS pattern was substantially higher among the minority population (7/347, 2.0%), which included Uygurs and Kazakhs than in Han Chinese (8/2,328, 0.3%, *P*<0.0001).

### Comparison of the Prevalence of the DFS Pattern in Adult and Pediatric Patients Versus Healthy Population Cohort

Quantitative comparisons between the RJ-PA and RJ-HEALTH cohorts, along with patient distributions in different departments and antibody titers, are shown in **Table 2** and **Supplementary Table 1**. It should be noted that the prevalence of the DFS pattern in HEp-2 IFA positive sera was significantly higher in pediatric (10.6%) compared to adult patients (3.3%, *P*<0.001) in the RJ-PA cohort, but no statistical difference was observed in the RJ-HEALTH cohort (7.8%, *P*=0.173). The same trend was observed when investigating association with gender in various populations in **Table 2**. DFS pattern was more common in females in the HEp-2 IFA screening cohort (females: 3.6%, 231/6,461, males: 1.6%, 16/1,029, *P*<0.0001), while no gender differences were observed in pediatric patients (females: 9.2%, 26/284, males: 17.2%, 11/64, *P*=0.060) or the healthy population (females: 6.3%, 17/268, males: 10.3%, 16/155, *P*=0.141). Moreover, the same distributions of DFS pattern titers were detected among adult and pediatric RJ-PA and RJ-HEALTH cohorts, as over 40% of titers were ≥1/640 (**Supplementary Table 1**).

**Table 2:** Comparison of the DFS pattern between adult and pediatric patient and healthy population cohorts by routine HEp-2 IFA in Renji Hospital during a 3-month study period.

	Routine HEp-2 IFA cohort (RJ-PA cohort)							Healthy population cohort (RJ-HEALTH cohort)			*P*^†^
	Total	Adult			Pediatric			Total	Females/Males	*P*	
		Total	Females/Males	*P*	Total	Females/Males	*P*				
HEp-2 IFA tested, n	21049	20293	15830/4463		756	497/259		4234	1753/2481		
IFA positive, n (%)	7838(37.2)	7490(36.9)	6461(40.8)/ 1029(23.1)^a^	<0.001	348(46.0)	284(57.1)/ 64(24.7)^a^	<0.001	423(10.0)	268(15.3)/ 155(6.2)^a^	<0.001	<0.001
DFS pattern in IFA positive, n (%)	284(3.6)	247(3.3)	231(3.6)/ 16(1.6)^b^	<0.001	37(10.6)	26(9.2)/ 11(17.2)^b^	NS	33(7.8)	17(6.3)/ 16(10.3)^b^	NS	<0.001
DFS pattern in HEp-2 IFA tested, %	1.3	1.2	1.5/0.4^c^		4.9	5.2/4.2^c^		0.8	1.0/0.6^c^		
Age (year, x ± SD)		39.4 ± 13.7			11.3 ± 3.4			43.6 ± 13.3			NS

a The percentage of HEp-2 IFA positive female/male patients in routine HEp-2 IFA screen testing cohort.

b The percentage of female/male patients with DFS pattern in HEp-2 IFA positive population.

c The percentage of female/male patients with DFS pattern in routine HEp-2 IFA screen testing cohort.

^†^The P value was compared between adults in RJ-PA cohort with the RJ-HEALTH cohort.

HEp-2 IFA, HEp-2 cell indirect immunofluorescence assay.

NS, no statistical significance.

In the RJ-PA cohort, the percentages of SARD patients with DFS pattern were compared respectively with HEp-2 IFA negative (AC-0) and three other common patterns including nuclear homogeneous (AC-1), fine speckled (AC-4), and large/coarse speckled (AC-5), and remaining patterns (**Table 3**). In the RJ-PA adult cohort, the percentage of SARD in each AC pattern was much lower in patients with DFS pattern compared to those with AC-1, AC-4, AC-5 or other remaining patterns, but higher than the HEp-2 IFA negatives. In contrast, the percentage of SARD in pediatric patients with DFS pattern was comparable to those with HEp-2 IFA negative or AC-1, lower than AC-4 or AC-5, and higher than other remaining patterns.

**Table 3:** Comparison of the percentages of SARD patients with DFS pattern (AC-2) versus with ANA-negative (AC-0) and other AC patterns in the RJ-PA adult and pediatric cohorts.

	AC-2	AC-0	AC-1	AC-4	AC-5	Other AC
Adult patients (n=20293)	n=247, 1.2%	n=12803, 63.1%	n=1633, 8.0%	n=3419, 16.8%	n=723, 3.6%	n=1468, 7.2%
SARD, n (%^a^/%^b^)	25 (0.1/10.1)	535 (2.6/4.2)	709 (3.5/43.4)	1256 (6.2/36.7)	469 (2.3/64.9)	315 (1.6/21.5)
*P*^c^		**<0.001**	**<0.001**	**<0.001**	**<0.001**	**<0.001**
Pediatric patients (n=756)	n=37, 4.9%	n=408, 54.0%	n=52, 6.9%	n=132, 17.5%	n=48, 6.3%	n=79, 10.4%
SARD, n (%^a^/%^b^)	18 (2.4/48.6)	199 (26.3/48.8)	35 (4.6/67.3)	108 (14.3/81.8)	40 (5.3/83.3)	12 (1.6/15.2)
*P*^c^		0.988	0.077	**<0.001**	**0.007**	**<0.001**

SARD, systemic autoimmune rheumatic disease; HEp-2 IFA, HEp-2 cell indirect immunofluorescence assay; DFS, dense fine speckled; AC, anti-cell; AC-2, anti-cell-2 pattern (DFS pattern); AC-1, nuclear homogeneous pattern; AC-4, nuclear fine speckled pattern; AC-5, nuclear large/coarse speckled pattern; AC-0, HEp-2 IFA negative.

Other AC, other anti-cell patterns except AC-0, AC-1, AC-2, AC-4, and AC-5.

a Percentages of SARD patients in adult or pediatric patients in routine HEp-2 IFA screen cohort.

b Percentages of SARD patients in respective HEp-2 IFA patterns.

c P value is the SARD patient positive rate in each AC pattern compared with that in AC-2 pattern. Bold characters indicate significant differences between two groups.

### Consistency of Interpretation Rates of the DFS Pattern Between Renji Hospital and Participating Centers

**Figure 3:**
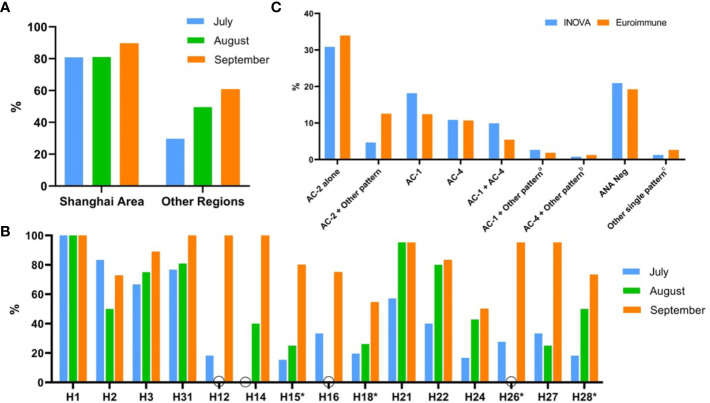
The apparent improvement (%) in correctly recognizing DFS pattern in many participating centers during the training program from July to September 2019. Putative DFS sera sent to the organizing center at Renji Hospital were re-analyzed and the accuracy of DFS pattern reporting was calculated as interpretation consistency rate. **(A)** Comparison of the interpretation consistency rate of DFS pattern by Renji Hospital versus 4 centers in Shanghai area and 25 centers in more remote provinces in China (Other Regions). **(B)** Apparent improvement in DFS pattern interpretation in representative hospitals with codes as indicated in Circles mark months when serum samples were not delivered to Renji Hospital. Centers with statistical differences showing improvement of their interpretation consistency rate between July and September were marked with asterisk. **(C)** Comparison of results in the re-testing of serum samples sent by the 29 centers to Renji Hospital using Inova and Euroimmun ANA HEp-2 cell slides. AC-2: dense fine speckled (DFS) pattern, AC-2 + Other pattern: DFS pattern mixed with other ANA pattern, AC-1: nuclear homogeneous pattern, AC-4: nuclear speckled pattern, Neg: ANA negative results. a: AC-1 mixed with another pattern (AC-1 + AC-4 not included); b: AC-4 mixed with another pattern (AC-1 + AC-4 not included); c: Other single patterns including AC-5 - nuclear large/coarse speckled, AC-8 - homogeneous nucleolar and AC-28 - mitotic chromosomal.

At the onset of this study, only 14 of the 29 participating laboratories routinely reported the DFS pattern (**Table 1**). Among the participating centers, the mean years of experience with reading ANA patterns was 4.4 ± 2.9 years. The interpretation consistency rates between Renji Hospital and other centers were low, as only 46.2% (298/645) of delivered sera were confirmed positive for the DFS pattern. Heterogeneity in medical resources might be a reason for the lack of expertise in the recognition of the DFS pattern in some centers. **Figure 3A** shows a relatively small change in interpretation consistency from 80.8% to 89.5% (*P*=0.535) in Shanghai centers from July to September, compared to a more obvious increase from 29.7% to 60.6% (*P*<0.0001) in other regions. An apparent improvement in DFS pattern interpretation was observed in many participating centers throughout the program (**Figure 3B**).

### Comparison of DFS Pattern Interpretation by Two Commercial HEp-2 IFA Kits

All 645 serum samples delivered from the participating centers were tested on two different commercial HEp-2 IFA slides to evaluate the agreement in identifying the DFS pattern. There was good agreement between the two brands (kappa=0.598) as 31.2% (201/645) of sera showed single or mixed DFS pattern on both slides, while 4.3% (28/645) and 15.3% (99/645) exhibited the DFS pattern only in Inova Diagnostics or Euroimmun slides, respectively. Some of the typical inconsistent cases are shown in **Supplementary Figure 1**. Moreover, 49.1% (317/645) of sera did not show the DFS pattern on either type of slide. All the misinterpreted patterns are listed in **Figure 3C**, which shows that the nuclear homogeneous pattern (AC-1) was the most difficult pattern to distinguish from DFS. 18.1% and 12.4% of sera were misinterpreted as nuclear homogeneous on Inova and Euroimmun slides, respectively.

### Anti-DFS70 Antibodies Detected by ELISA, LIA, and HEp-2 ELITE

All 645 sera interpreted as single or mixed DFS pattern on Inova or Euroimmun HEp-2 slides were further analyzed by ELISA, LIA, and IFA on HEp-2 ELITE/DFS KO substrate. Sera with DFS pattern on both Inova and Euroimmun slides were significantly more likely to be positive for anti-DFS70 antibody in ELISA, LIA, or HEp-2 ELITE assays than sera showing positive in only one of the slides. Anti-DFS70 positive rates among the samples positive on both versus only one commercial slide were 96.0% (457/476) and 63.3% (107/169) (*P*<0.0001) by ELISA, 93.7% (446/476) and 56.8% (96/169) (*P*<0.0001) by LIA, and 49.6% (236/476) and 29.6% (50/169) (*P*<0.0001) by HEp-2 ELITE, respectively (**Figure 2B**). Anti-DFS70 reference material was tested by HEp-2 ELITE/DFS KO substrate as a positive control (**Supplementary Figure 2**). In our in-house anti-DFS70 ELISA, 60 samples negative for anti-DFS70, confirmed by LIA, were used to establish a cutoff of 0.6 (mean O.D. value of 0.214 + 3 × 0.130 SD). The O.D. value for anti-DFS70 reference material was 2.240. The correlations measured between ELISA O.D. value with HEp-2 IFA titer (Spearman r=0.501, *P*<0.0001) and NI read by NOVA VIEW 1.0 (Spearman r=0.506, *P*<0.0001) were considered significant (**Supplementary Figure 3**).

### Clinical Phenotypes and Additional ENA Specificities in Anti-DFS70 Positive Sera

In total, 84.7% (546/645) of serum samples with the DFS pattern, including 517 samples from the HEp-2 IFA screening cohort and 29 sera from the RJ-HEALTH cohort, showed positive reactivity to DFS70 by both ELISA and LIA. Of these positive patients, 336 adult (89.4%) and 40 pediatric (10.6%) patients had traceable clinical phenotypes. The spectrum of disease and clinical conditions of adult and pediatric patients with positive reactivity to DFS70 by both ELISA and LIA were analyzed respectively in **Table 4** and the disease groups were detailed in **Supplementary Table 2**. For SARD group, the most prevalent diseases among DFS70-positive adult and pediatric patients were spontaneous abortion (28.0%, 94/336) and juvenile idiopathic arthritis (JIA, 22.5%, 9/40), respectively (**Supplementary Table 2**). In investigating anti-DFS70 positive patients with SARD, half of the anti-DFS70 positive pediatric patients were diagnosed with SARD including 9 JIA (22.5%, 9/40), 8 undifferentiated connective tissue disease (UCTD) (20.0%, 8/40) and 3 SLE (7.5%, 3/40) while anti-DFS70 positive. In contrast, adult patients included 28 UCTD (8.3%, 28/336), 13 rheumatoid arthritis (3.9%, 13/336), 5 SLE (1.5%, 5/336), 5 Sjogren’s syndrome (1.5%, 5/336) and 1 antiphospholipid syndrome (0.3%, 1/336).

**Table 4:** Clinical diagnoses of adult and pediatric patients with positive reactivity to DFS70 by both ELISA and LIA.

Disease	Adult	Child
Systemic autoimmune disease	52 (15.5)	20 (50.0)
Organ-specific autoimmune disease	3 (0.9)	1 (2.5)
Inflammatory diseases	25 (7.4)	8 (20.0)
Gynecologic syndromes	118 (35.1)	0 (0.0)
Neoplasms	15 (4.5)	0 (0.0)
Infectious diseases	12 (3.6)	2 (5.0)
Miscellaneous	111 (33.0)	9 (22.5)
Total	336	40

DFS70, dense fine speckled 70 kDa antigen; ELISA, enzyme-linked immunosorbent assay; LIA, line immunoblot assay.

All 376 patients were further tested for additional anti-ENA antibodies by LIA. The prevalence of “monospecific” anti-DFS70 was higher in non-SARD patients (76.8%, 289/376) than in SARD patients (15.2%, 57/376; *P*<0.0001) as shown in **Supplementary Table 3**. The additional anti-ENA positive rates were as follows: anti-Ro52/TRIM21 4.8% (SARD: 9.7%, non-SARD: 3.7%, *P*=0.059), anti-SSA/Ro60 2.7% (SARD: 6.9%, non-SARD: 1.7%, *P*=0.027), anti-Rib-P 1.1% (SARD: 4.2%, non-SARD: 0.3%, *P*=0.024), anti-histone 1.1% (SARD: 2.8%, non-SARD: 0.7%, *P*=0.170), anti-nRNP/Sm 0.8% (SARD: 4.2%, non-SARD: 0.0%, *P*=0.007), and anti-SSB/La 0.3% (SARD: 1.4%, non-SARD: 0.0%, *P*=0.194). The details of additional ENAs in adult and pediatric patients with anti-DFS70 in different clinical diagnoses are listed in **Supplementary Table 4**. Moreover, additional anti-ENAs were not detected in DFS sera from the RJ-HEALTH cohort.

## Discussion

A large-scale, short-term multi-center study was conducted with 30 laboratories from various regions in China, of which 16 started with no experience of reporting DFS pattern and 14 had begun reporting DFS pattern only one or two years prior to the study. After the DFS pattern training/re-orientation program, we found that: 1) the short-term training greatly improved DFS pattern recognition in many of the participating laboratories as the interpretation consistency increased from 29.7 to 60.6% in the areas other than Shanghai, 2) the pattern identification consistency was higher in areas with well-developed health facilities (i.e. the consistency rate in the four laboratories in Shanghai was 80.8 versus 29.7% for the other participating laboratories at the start of the program), 3) sera with DFS pattern confirmed by two commercial HEp-2 slides showed higher positive rates for anti-DFS70 antibodies than by only one slide brand (96.0 versus 56.2, or 75.0%).

To better understand the prevalence of DFS pattern in the routine HEp-2 IFA screening cohort of this study, we have summarized HEp-2 IFA positive rates from different reports worldwide in **Supplementary Table 5**. The reported HEp-2 IFA positive rates in **Supplementary Table 5** based on routine screen test cohorts range from 11.6–82.0% with a median of 28.5%, which is close to our present study of 36.2%. However, our prevalence of the DFS pattern was 0.6% in the routine HEp-2 IFA screening cohort, which is relatively low compared with reports from other countries (**Supplementary Table 5**). There are a few possible explanations for this low frequency. First, ethnicity may have played a role in the prevalence of the DFS pattern/anti-DFS70. To put this into perspective, the highest prevalence of 27% was only reported in the United States (16) compared to 0.3–8.4% reported in other continents (**Supplementary Table 5**). In the present study, the HEp-2 IFA positive rate was significantly higher for Han Chinese than for Uygurs and Kazakhs, yet DFS pattern positive rates were higher among these minority populations. In contrast, the HEp-2 IFA positive rate was higher in Tibetans, Huis, and Mongolians than Han Chinese, while the DFS pattern positive rate in HEp-2 IFA positive sera showed no statistical difference. To our knowledge, this is the first report to compare HEp-2 IFA positive rates between national minority groups and Han Chinese in China. Second, reported DFS pattern positive rates can also be affected by the experience of IFA evaluators (16, 23). In **Supplementary Table 5**, the reported prevalence of the DFS pattern in Turkey varied widely from 0.3 to 8.1% (24). In North America, the prevalence of DFS has been reported as 27.0% in the United States (16) but only 1.6% in Canada (6). As the present study shows, improving the accuracy of DFS pattern interpretation in some of the participating centers, especially those in areas with relatively poor medical resources, will affect the overall reported anti-DFS70 prevalence. Finally, application of different commercial HEp-2 IFA kits may contribute to the discrepancy in prevalence of the DFS pattern (25, 26). Due to the inevitable heterogeneity in performance of various slides, the consistency of the two kits used in our study only exhibited moderate agreement (kappa=0.598) in DFS pattern identification.

We further investigated the prevalence of the DFS pattern in pediatric patients. Schmeling et al. (27) reported a 4.5% positive rate (titer ≥1:80) of anti-DFS70 in pediatric patients referred for ANA testing, which is comparable to the 4.9% reported in the present study. Anti-DFS70 antibodies have been reported to be more prevalent in younger age groups (4, 28). This may partly explain why the positive rate of DFS pattern was higher in pediatric versus adult patients in this study. However, no statistical difference between pediatric patients and adult healthy individuals was found. Notably, the DFS pattern was more prevalent in adult female patients (females 3.6%, males 1.6%), while no gender differences were observed among the pediatric patient and healthy populations. Therefore, the prevalence features of anti-DFS70 in pediatric patients were similar to those in the healthy adult population. To date, few studies have focused on anti-DFS70 in pediatric patients. The prevalence in healthy children should be further investigated and compared to pediatric patients in the same age category (29).

As accurate DFS pattern interpretation by IFA alone is challenging, additional objective tests such as ELISA, LIA, or chemiluminescence immunoassay (CLIA) are necessary to identify anti-DFS70 antibodies (25, 30–32). ELITE/DFS KO substrate also offers a unique possibility of evaluating anti-DFS70 antibodies at the IFA stage (23, 33). Reports have shown that ELITE/DFS KO substrate can improve the sensitivity of confirming anti-DFS70 antibodies to 65% compared to 61% by CLIA (23). In our study, the sensitivity of ELITE/DFS KO substrate was far below both ELISA and LIA. Thus, we recommend clinical laboratories apply anti-DFS70 antibody methodologies like ELISA and LIA instead of ELITE/DFS KO substrate.

Carter et al. reported 73.1% monospecific DFS70 in anti-DFS70 positive sera from a HEp-2 IFA screening cohort which was lower than our findings of 92.0%. Monospecific anti-DFS70 is considered rare in SARD and may serve as an exclusion biomarker for SARD (5, 7, 34). In a multi-center study, Choi et al. reported 1.1% monospecific anti-DFS70 in a large cohort of SLE patients (34). In our study, the percentage of monospecific anti-DFS70 patients was much higher in non-SARD (76.8%) than in SARD (15.2%) patients in HEp-2 IFA screening cohort. This to some extent supports that monospecific anti-DFS70 is a reliable biomarker to rule out diagnosis of SARD (35). Moreover, anti-Ro52/TRIM21 and anti-SSA/Ro60 were the most commonly detected autoantibodies accompanying anti-DFS70 antibodies in our LIA ENA profiles, while SLE-specific antibodies including Sm and dsDNA were not observed. Choi et al. (34) also reported anti-SSA/Ro60 (34.6%) and anti-Ro52/TRIM21 (27.2%) detected by addressable laser bead immunoassay array, as the most common autoantibodies found with anti-DFS70 antibodies in SLE patients, which is consistent with our results.

Regarding the clinical relevance of anti-DFS70 antibodies, there were some unexpected findings. First, the effect of DFS pattern in helping to rule out SARD in the routine HEp-2 IFA screening cohort was not in fact stronger than negative ANA in adults, while no statistical difference was observed between DFS pattern and negative ANA in pediatric patients. The latter can be explained partly to the high percentage of JIA patients (26.6%, 201/756) in the pediatric ANA screen cohort and the HEp-2 IFA positive rate of JIA was 21.9% (44/201) (data not shown). Moreover, it is still worth noting that the frequency of SARD in anti-DFS70 positive pediatric patients was unexpectedly as high as 50.0%. We consider this data significant because in this RJ-PA pediatric cohort, the percentage of SARD patients with DFS pattern was similar to those with AC-1 and much higher than those with other ANA patterns except AC-4 and AC-5. However, in the RJ-PA adult cohort, the percentage of SARD patients with DFS pattern was much lower than those with AC-1, AC-4, AC-5 or even those with other patterns. Together, this data strongly suggests that anti-DFS70 prevalence was different in pediatric and adults patients with SARD. Sperotto et al. tested four monospecific anti-DFS70 positive cases out of a population of 261 school-age children and found that three of the cases (75%) had a family history of autoimmune disease, but no disease symptoms (29). Moreover, none of the ANA-positive (anti-DFS70 positive/negative) subjects developed SARD in a three-year follow up (29). Therefore, future multi-center studies should focus on the underlying role of anti-DFS70 in pediatric cohorts. Second, the proportion of anti-DFS70 positive adult patients who had experienced spontaneous abortion from the ANA screening cohort was remarkably high at 28.0%. The highest rate of DFS pattern among the HEp-2 IFA positive population was observed in an obstetrics and gynecology hospital in Shanghai, China. For the first time, these data suggest that anti-DFS70 antibodies may be associated with female reproductive diseases. Furthermore, in an ongoing study in the Renji Hospital, the prevalence of DFS pattern in females with spontaneous abortion (3.1%, 94/2990) was much higher than age-matched healthy females (1.5%, 45/2990; *P*<0.0001, data not shown), which also support the association between them. Isolated studies have also reported anti-DFS70 in eye diseases, like sympathetic ophthalmia (9), Vogt-Koyanagi-Harada syndrome (11), and atopic dermatitis with cataracts (36), and as tumor-associated antibodies present in prostate cancer patients (8, 13, 37). Marlet et al. (38) was the first to report that anti-DFS70 antibodies may be correlated with thrombosis and obstetric complications. DFS70 has been shown to be upregulated by human papilloma virus (HPV) infection and its associated oncogenes E6/E7 (39) and has been implicated in autoimmune thyroiditis (40). Both diseases are also associated with several reproductive pathology (41). However, anti-DFS70 have previously been shown to be associated with young age and female sex (40). Therefore, to better investigate the association between spontaneous abortions and anti-DFS70, the prevalence of anti-DFS70 needs to be compared between the pregnant women who completed the pregnancy versus those with spontaneous abortions. Further studies are necessary to explore the clinical association between anti-DFS70 and reproductive diseases.

In addition to those mentioned above, UCTD and RA were the most common SARD in adults associated with anti-DFS70, while JIA, UCTD, and pediatric SLE were seen in anti-DFS70 positive pediatric patients. This is consistent with some previous reports that anti-DFS70 antibodies may be restricted to SARD patients without typical ANA-associated antibodies, and only rarely found in patients with ANA-associated rheumatic disease (6, 7, 42). Infantino et al. also reported high prevalence of anti-DFS70 antibodies in UCTD cases (13.3%) and suggested that anti-DFS70 antibodies could serve as an appropriate biomarker for the development of UCTD to CTD (28).

One limitation of this study was that we were unable to obtain some additional laboratory test results, including anti-thyroid peroxidase antibodies, serum free triiodothyronine, thyroxine, thyroid-stimulating hormone, and activated partial thromboplastin time for the anti-DFS70 positive cohort from the participating laboratories to further explore their relationship to anti-DFS70 positive patients.

In conclusion, DFS pattern interpretation can be a challenging task for many clinical laboratories. However, a short-term training course and inter-laboratory comparison of HEp-2 IFA results can improve IFA pattern reading accuracy. The prevalence of ANA and the DFS pattern may vary between different Chinese ethnic groups. The clinical usefulness of anti-DFS70 may help to exclude SARD in adult patients. The increased prevalence of spontaneous abortion and pediatric SARD in anti-DFS70 positive patients will require further follow-up studies.

## Data Availability Statement

The original contributions presented in the study are included in the article/**Supplementary Material**; further inquiries can be directed to the corresponding authors.

## Ethics Statement

All serum specimens collected in this study were approved by the Institutional Review Board of Renji Hospital (No. KY[2019]121). No consent was required for this study.

## Author Contributions

BZ, ZW, AL, CL, DL, FZ, HL, HG, JZ, JL, LC, LW, LY, LNY, LJ, MFH, MH, PX, QL, SH, SSC, SMC, SZ, WS, XG, YC, YW, YQ, ZL, ZN, and ZH assisted HEp-2 IFA pattern interpretation. ZW performed the experiments. BZ analyzed the experiments and wrote the manuscript. EC supervised the study and revised the manuscript. BZ and RM read and revised the manuscript. All authors contributed to the article and approved the submitted version.

## Funding

This work was supported by Shanghai Medical and Health Development Foundation grant 2019 and National Natural Science Foundation of China (grants 81772139). EC is supported by NIH grant R01DE028536.

## Conflict of Interest

The authors declare that the research was conducted in the absence of any commercial or financial relationships that could be construed as a potential conflict of interest.

The handling editor declared a past co-authorship with one of the authors EC.

## Abbreviations

ANA, antinuclear antibodies; AC-2, anti-cell-2; DFS70, dense fine speckled 70 kDa antigen; CLIA, chemiluminescence immunoassay; CTD, connective tissue disease; ELISA, enzyme-linked immunosorbent assay; ENA, extractable nuclear antigen; HEp-2 IFA, HEp-2 cell indirect immunofluorescence assay; ICAP, International Consensus on Antinuclear Antibody Patterns; LIA, line immunoblot assay; NI, nuclear intensities; O.D., optical density; Rib-P, ribosomal P proteins; RA, rheumatoid arthritis; SARD, systemic autoimmune rheumatic disease; SD, standard deviations; UCTD, undifferentiated connective tissue disease.
