# Supplementary material for: Anti-DFS70 Antibodies Among Patient and Healthy Population Cohorts in China: Results From a Multicenter Training Program Showing Spontaneous Abortion and Pediatric Systemic Autoimmune Rheumatic Diseases Are Common in Anti-DFS70 Positive Patients
Source: Front Immunol. 2020 Oct 2;11:562138. doi: 10.3389/fimmu.2020.562138 (PMC7566153; doi:10.3389/fimmu.2020.562138)
Supplement: Supplementary file 1 [file DataSheet_1.docx]

**Supplementary materials for ‘Anti-DFS70 antibodies among patient and healthy population cohorts in China: results from a multicenter training program showing spontaneous abortion and pediatric systemic autoimmune rheumatic diseases are common in anti-DFS70 positive patients’**

**Supplementary Tables and Figures**

**Supplementary Table 1.** Characteristics of DFS pattern between routine ANA adult and pediatric patient and healthy population cohorts in Renji Hospital during a 3-month study period.

|  | Routine HEp-2 IFA cohort  (RJ-PA cohort) | | |  | Healthy population cohort  (RJ-PE cohort) |
| --- | --- | --- | --- | --- | --- |
|  | Total | Adult | Pediatric |  |  |
| DFS pattern titer, n (%) | | | |  |  |
| 1/80 | 49 (17.3) | 44 (17.8) | 5 (13.5) |  | 4 (12.1) |
| 1/160 | 65 (22.9) | 62 (25.1) | 3 (8.1) |  | 7 (21.2) |
| 1/320 | 51 (18.0) | 39 (15.8) | 12 (32.4) |  | 9 (27.3) |
| 1/640 | 83 (29.2) | 70 (28.3) | 13 (35.2) |  | 9 (27.3) |
| 1/1280 | 36 (12.7) | 32 (13.0) | 4 (10.8) |  | 4 (12.1) |
| Department distribution of DFS pattern, n (%) | | | |  |  |
| Rheumatology | 121 (42.6) | 110 (44.5) | 11 (29.7) |  |  |
| Gynecology | 96 (33.8) | 96 (38.9) | 0 (0.0) |  |  |
| Pediatrics | 26 (9.2) | 0 (0.0) | 26 (70.3) |  |  |
| Gastroenterology | 12 (4.2) | 12 (4.9) | 0 (0.0) |  |  |
| Hematology | 11 (3.9) | 11 (4.5) | 0 (0.0) |  |  |
| Nephrology | 8 (2.8) | 8 (3.2) | 0 (0.0) |  |  |
| Other departments | 10 (3.5) | 10 (4.0) | 0 (0.0) |  |  |
| Anti-DFS70 antibodies | | | |  |  |
| ELISA | 272 (95.8) | 235 (95.1) | 37 (100) |  | 30 (90.9) |
| LIA | 269 (94.7) | 233 (94.3) | 36 (97.3) |  | 31 (93.9) |
| HEp-2 ELITE | 137 (48.2) | 121 (49.0) | 16 (43.2) |  | 11 (33.3) |

HEp-2 IFA: HEp-2 cell indirect immunofluorescence assay; ELISA: enzyme-linked immunosorbent assay, LIA: line immunoblot assay, HEp-2 ELITE: immunofluorescence assay on HEp-2 ELITE/DFS70-KO substrate.

**Supplementary Table 2.** Clinical diagnosis of patients with positive reactivity to DFS70 by both ELISA and LIA assay.

| Disease | Adult | |  | Child | |
| --- | --- | --- | --- | --- | --- |
|  | No. of patients | Frequency (%) |  | No. of patients | Frequency (%) |
| **Systemic autoimmune disease** |  | 15.5 |  |  | 50.0 |
| Undifferentiated CTD | 28 | 8.3 |  | 8 | 20.0 |
| Rheumatoid arthritis | 13 | 3.9 |  | 0 | 0.0 |
| Systemic lupus erythematosus | 5 | 1.5 |  | 3 | 7.5 |
| Sjogren's syndrome | 5 | 1.5 |  | 0 | 0.0 |
| Juvenile idiopathic arthritis | 0 | 0.0 |  | 9 | 22.5 |
| Antiphospholipid syndrome | 1 | 0.3 |  | 0 | 0.0 |
|  |  |  |  |  |  |
| **Organ-specific autoimmune disease** |  | 0.9 |  |  | 2.5 |
| Autoimmune hepatitis | 2 | 0.6 |  | 0 | 0.0 |
| Autoimmune thyroiditis | 1 | 0.3 |  | 0 | 0.0 |
| Immune thrombocytopenic purpura | 0 | 0.0 |  | 1 | 2.5 |
|  |  |  |  |  |  |
| **Inflammatory diseases** |  | 7.4 |  |  | 20.0 |
| Arthritis | 8 | 2.4 |  | 0 | 0.0 |
| Vasculitis | 4 | 1.2 |  | 2 | 5.0 |
| Urticaria | 3 | 0.9 |  | 0 | 0.0 |
| Anaphylactoid purpura | 1 | 0.3 |  | 3 | 7.5 |
| Iridocyclitis | 0 | 0.0 |  | 2 | 5.0 |
| Alopecia areata | 0 | 0.0 |  | 1 | 2.5 |
| Ulcerative colitis | 1 | 0.3 |  | 0 | 0.0 |
| Crohn's disease | 1 | 0.3 |  | 0 | 0.0 |
| Adult-onset Still disease | 1 | 0.3 |  | 0 | 0.0 |
| Dermatomyositis | 1 | 0.3 |  | 0 | 0.0 |
| Gastroduodenal ulcer | 1 | 0.3 |  | 0 | 0.0 |
| Anterior uveitis | 1 | 0.3 |  | 0 | 0.0 |
| Oral aphthae ulceration | 1 | 0.3 |  | 0 | 0.0 |
| Takayasu arteritis | 1 | 0.3 |  | 0 | 0.0 |
| Fasciitis | 1 | 0.3 |  | 0 | 0.0 |
|  |  |  |  |  |  |
| **Gynecologic syndromes** |  | 35.1 |  |  | 0.0 |
| Spontaneous abortion | 94 | 28.0 |  | 0 | 0.0 |
| Female infertility | 16 | 4.8 |  | 0 | 0.0 |
| Pregnancy | 5 | 1.5 |  | 0 | 0.0 |
| Pregnancy with other diseases ^a^ | 3 | 0.9 |  | 0 | 0.0 |
|  |  |  |  |  |  |
| **Neoplasms** |  | 4.5 |  |  | 0.0 |
| Multiple myeloma | 9 | 2.7 |  | 0 | 0.0 |
| Lymphoma | 2 | 0.6 |  | 0 | 0.0 |
| Hysteromyoma | 2 | 0.6 |  | 0 | 0.0 |
| Duodenum cancer | 1 | 0.3 |  | 0 | 0.0 |
| Prostate cancer | 1 | 0.3 |  | 0 | 0.0 |
|  |  |  |  |  |  |
| **Infectious diseases** |  | 3.6 |  |  | 5.0 |
| Hepatitis B | 6 | 1.8 |  | 0 | 0.0 |
| Pneumonia | 3 | 0.9 |  | 1 | 2.5 |
| Urinary tract infection | 2 | 0.6 |  | 0 | 0.0 |
| Tubercle bacilli | 1 | 0.3 |  | 0 | 0.0 |
| Epstein-Barr virus | 0 | 0.0 |  | 1 | 2.5 |
|  |  |  |  |  |  |
| **Miscellaneous** |  | 33.0 |  |  | 22.5 |
| Checkup | 42 | 12.5 |  | 6 | 15.0 |
| Joint pain | 39 | 11.6 |  | 0 | 0.0 |
| Proteinuria | 6 | 1.8 |  | 1 | 2.5 |
| Chronic kidney disease | 6 | 1.8 |  | 0 | 0.0 |
| Baldness | 5 | 1.5 |  | 0 | 0.0 |
| Coronary atherosclerosis | 3 | 0.9 |  | 0 | 0.0 |
| Hypertension | 3 | 0.9 |  | 0 | 0.0 |
| Diabetes mellitus | 2 | 0.6 |  | 0 | 0.0 |
| Rheumatic heart diseases | 1 | 0.3 |  | 0 | 0.0 |
| Allergic contact dermatitis | 1 | 0.3 |  | 0 | 0.0 |
| Chronic obstructive pulmonary disease | 1 | 0.3 |  | 0 | 0.0 |
| Nephrotic syndrome | 1 | 0.3 |  | 1 | 2.5 |
| Gout | 1 | 0.3 |  | 0 | 0.0 |
| Hyperuricemia | 0 | 0.0 |  | 1 | 2.5 |

^a^ Pregnancy with cervical insufficiency, Hashimoto's thyroiditis, and hyperthyroidism respectively.

CTD, connective tissue disease.

**Supplementary Table 3.** Monospecific anti-DFS70 antibodies are primarily detected in non-SARD patients.

| Anti-DFS70 positive cases  n=376 | | | | Total |
| --- | --- | --- | --- | --- |
| Monospecific anti-DFS70  92.0% | | Anti-DFS70 plus other anti-ENAs  8.0% | | 100% |
| Non-SARD  76.8% | SARD  15.2% | Non-SARD  4.0% | SARD  4.0% | 100% |

DFS70: dense fine speckled 70 kDa antigen; ENA: extractable nuclear antigen; SARD: systemic autoimmune rheumatic disease.

**Supplementary Table 4.** Additional ENAs in adult and pediatric patients with anti-DFS70 in different clinical diagnosis groups.^*^

|  | Adult | |  | Child | |
| --- | --- | --- | --- | --- | --- |
|  | Additional ENA  Frequency (%) | ENA (n) |  | Additional ENA  Frequency (%) | ENA (n) |
| Systemic autoimmune disease | 12/52 (23.1) | nRNP/Sm (2), SSA/Ro60 (5), Ro-52/TRIM 21 (7), SSB/La (1), Rib-P (3) |  | 3/20 (15.0) | nRNP/Sm (1), histones (2) |
| Organ-specific autoimmune disease | 0/3 (0.0) | - |  | 0/1 (0.0) | - |
| Non-Systemic autoimmune disease |  |  |  |  |  |
| Inflammatory diseases | 2/25 (8.0) | SSA/Ro60 (1), Ro-52/TRIM 21 (2) |  | 2/8 (25.0) | histones (2) |
| Gynecologic syndromes | 4/118 (3.4) | SSA/Ro60 (2), Ro-52/TRIM 21 (3) |  | 0/0 (0.0) | - |
| Neoplasms | 0/15 (0.0) | - |  | 0/0 (0.0) | - |
| Infectious diseases | 0/12 (0.0) | - |  | 0/2 (0.0) | - |
| Miscellaneous | 7/111 (6.3) | SSA/Ro60 (2), Ro-52/TRIM 21 (6), Rib-P (1) |  | 0/9 (0.0) | - |
| Total | 25/336 (7.4) |  |  | 5/40 (12.5) |  |

^*^ Anti-DFS70 defined by both ELISA and LIA assays.

**Supplementary Table 5.** Prevalence of DFS pattern and anti-DFS70 antibodies in routine ANA referral cohorts.

| Continent | Country | Total patient analyzed (n) | ANA positive  n (%) | IFA cutoff | DFS pattern n (%)^a^ | DFS pattern in ANA positive serum (%) | Anti-DFS70 in DFS pattern  n (%)/method | References |
| --- | --- | --- | --- | --- | --- | --- | --- | --- |
| **Europe** |  |  |  |  |  |  |  |  |
|  | Italy | 21,516 | ND | 1:40 | 172 (0.8) | ND | ND | (1) |
|  | Italy | 3,175 | 1,191 (37.5) | 1:160 | 120 (3.8) | 10.1 | 55 (45.8)/IB | (2) |
|  | Italy | 1,005 | ND | 1:80 | 22 (2.2) | ND | 19 (86.4)/HEp-2 Elite | (3) |
| **North America** | |  |  |  |  |  |  |  |
|  | Canada | 3,263 | ND | 1:80 | 53 (1.6) | ND | 52 (98.1)/CIA  53 (100)/ELISA | (4) |
|  | USA | 6,511 | 5,339 (82.0) | 1:40 | 1,758 (27.0) | 32.9 | 720 (41.0)/LIA | (5) |
| **South America** | |  |  |  |  |  |  |  |
|  | Brazil | 7,733 | 2,168 (28.0) | 1:80 | 650 (8.4) | 30.0 | ND | (6) |
|  | Brazil | 2,788 | 790 (28.3) | 1:40 | 57 (2.0) | 7.2 | ND | (7) |
| **Asia** |  |  |  |  |  |  |  |  |
|  | South Korea | 10,528 | ND | 1:100 | 181 (1.7) | ND | 109 (60.2)/ELISA | (8) |
|  | South Korea | 2,654 | 352 (13.3) | 1:40 | 101 (3.8) | 28.7 | ND | (9) |
|  | South Korea | 5,509 | 639 (11.6) | 1:80 | 125 (2.3) | 19.6 | 75 (60.0)/ELISA | (10) |
|  | Turkey | 5,800 | 1,302 (22.4) | 1:100 | 16 (0.3) | 1.2 | 0 (0.0)/LIA | (11) |
|  | Turkey | 3,432 | 1,968 (57.3) | 1:100 | 277 (8.1) | 14.1 | 187 (67.5)/LIA | (12) |
|  | China | 5,961 | 2,507 (42.1) | 1:100 | 58 (1.0) | 2.3 | ND | (13) |
|  | China | 95,131 | 34,417 (36.2) | 1:80 | 513 (0.5) | 1.5 | 473 (92.2)/ELISA  459 (89.5)/LIA  244 (47.6)/HEp-2 Elite | Present study^b^ |
|  |  |  |  | 1:100 | 552 (0.6) | 1.6 | 488 (88.4)/ELISA  461 (83.5)/LIA  239 (43.3)/HEp-2 Elite | Present study^c^ |
| **Australia** |  |  |  |  |  |  |  |  |
|  | Australia | 181,819 | 51,905 (28.5) | 1:80 | 2,967 (1.6) | 5.7 | ND | (14) |

ND: no data; IB: immunoblot; CIA: chemiluminescence assay; ELISA: Enzyme-Linked Immunosorbent Assay; LIA: line immunoassay.

^a^ Ratio of DFS pattern in all routine ANA referral cohort.

^b^ HEp-2 IFA based on Inova slides.

^c^ HEp-2 IFA based on Euroimmun slides.

References

1. Bizzaro N, Tonutti E, Visentini D, Alessio MG, Platzgummer S, Morozzi G, et al. Antibodies to the Lens and Cornea in Anti‐DFS70‐Positive Subjects. 2007;1107(1):174-83.

2. Carbone T, Pafundi V, Tramontano G, Gilio M, Padula MC, Padula AA, et al. Prevalence and serological profile of anti-DFS70 positive subjects from a routine ANA cohort. 2019;9(1):1-7.

3. Infantino M, Shovman O, Gilburd B, Manfredi M, Grossi V, Benucci M, et al. Improved accuracy in DFS pattern interpretation using a novel HEp-2 ELITE system. 2019;38(5):1293-9.

4. Mahler M, Parker T, Peebles CL, Andrade LE, Swart A, Carbone Y, et al. Anti-DFS70/LEDGF antibodies are more prevalent in healthy individuals compared to patients with systemic autoimmune rheumatic diseases. 2012;39(11):2104-10.

5. Carter JB, Carter S, Saschenbrecker S, Goeckeritz BEJFiM. Recognition and relevance of anti-DFS70 autoantibodies in routine antinuclear autoantibodies testing at a community hospital. 2018;5:88.

6. Dellavance A, Viana VS, Leon EP, Bonfa ES, Andrade LE, Leser PGJTJor. The clinical spectrum of antinuclear antibodies associated with the nuclear dense fine speckled immunofluorescence pattern. 2005;32(11):2144-9.

7. Pazini AM, Fleck J, dos Santos RS, Beck STJRBR. Clinical relevance and frequency of cytoplasmic and nuclear dense fine speckled patterns observed in ANA-HEp-2. 2010;50(6):655-60.

8. Lee H, Kim Y, Han K, Oh EJSjor. Application of anti-DFS70 antibody and specific autoantibody test algorithms to patients with the dense fine speckled pattern on HEp-2 cells. 2016;45(2):122-8.

9. Kang SY, Lee W-IJTKjolm. Clinical significance of dense fine speckled pattern in anti-nuclear antibody test using indirect immunofluorescence method. 2009;29(2):145-51.

10. Koo SH, Kim J, Kim SY, Kwon GCJJoLM. Clinical significance of anti-DFS70 antibody in antinuclear antibody-positive samples with a dense fine speckled pattern. 2019;43(3):149-55.

11. Şener AG, Afşar İJEjor. Frequency of dense fine speckled pattern in immunofluorescence screening test. 2015;2(3):103.

12. Yumuk Z, Demir MJJoIM. Clinical value of anti-DFS70 antibodies in a cohort of patients undergoing routine antinuclear antibodies testing. 2020:112754.

13. Deng C, Qu X, Cheng S, Zeng X, Li Y, Fei YJAotrd. Decision-making value of nuclear dense fine speckled pattern in systemic autoimmune rheumatic disease: trick or treat? 2019.

14. Broadfoot A, Sivertsen T, Baumgart KJP. Dense fine speckled indirect immunofluorescence pattern in an Australian population. 2016;48(3):247-50.

**Supplementary Figure 1.** Three illustrative cases showing fine differences in IFA results by Inova and Euroimmun HEp-2 slides.


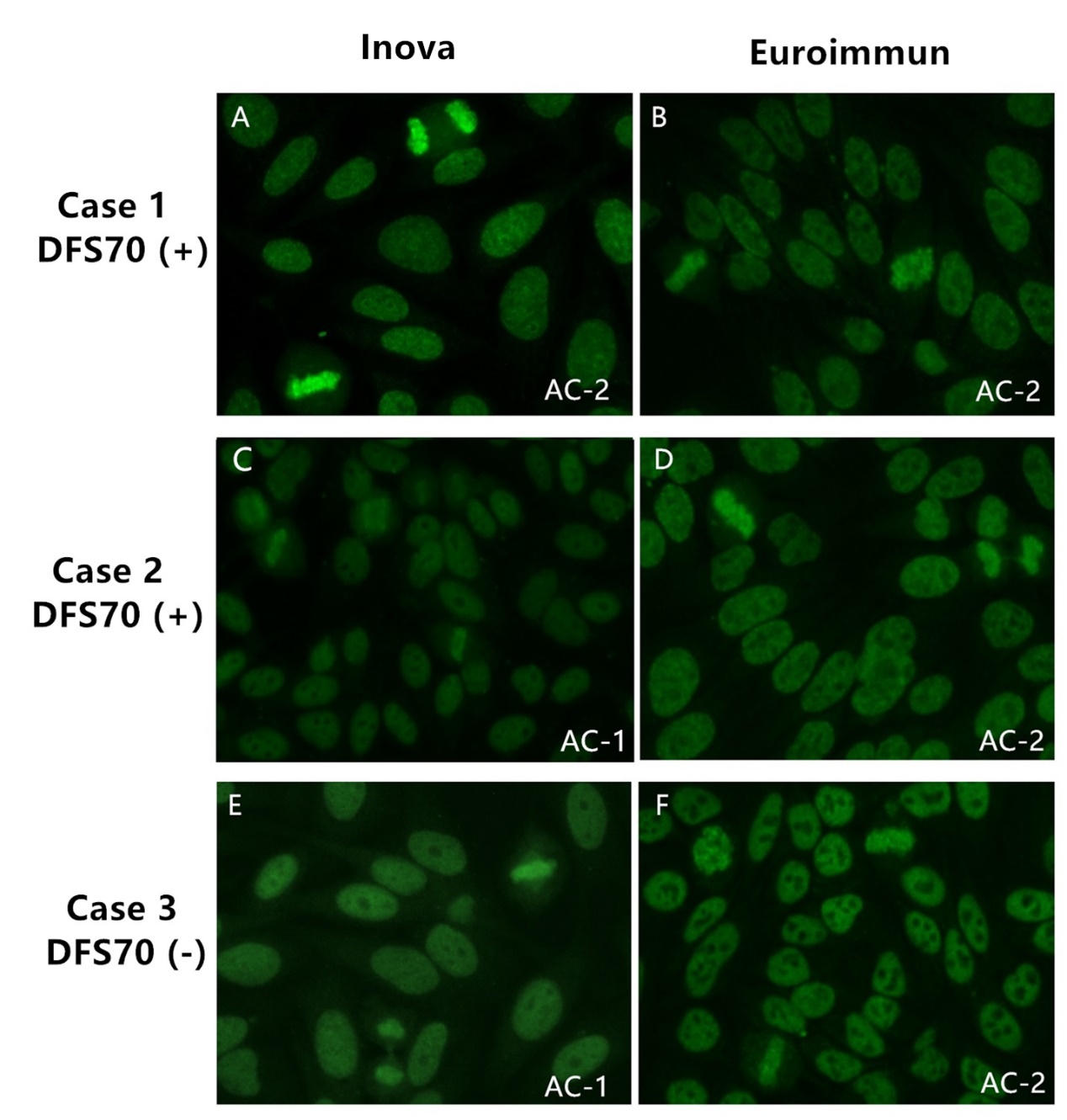


Case 1 presented DFS pattern (AC-2) in both slides and was identified as anti-DFS70 positive. Case 2 was anti-DFS70 positive and presented homogeneous pattern (AC-1) by Inova slide versus DFS pattern by Euroimmun slide. Case 3 was anti-DFS70 negative and exhibited homogeneous pattern (AC-1) and weak spindle fibers pattern (AC-25) by Inova slide versus DFS pattern by Euroimmun slide. All images were at the magnification of 400x. Sera were diluted 1:80 for Inova and 1:100 for Euroimmun slides.

**Supplementary Figure 2.** HEp-2 IFA patterns of the anti-DFS70 reference material for ICAP pattern AC-2 using different commercial slides.


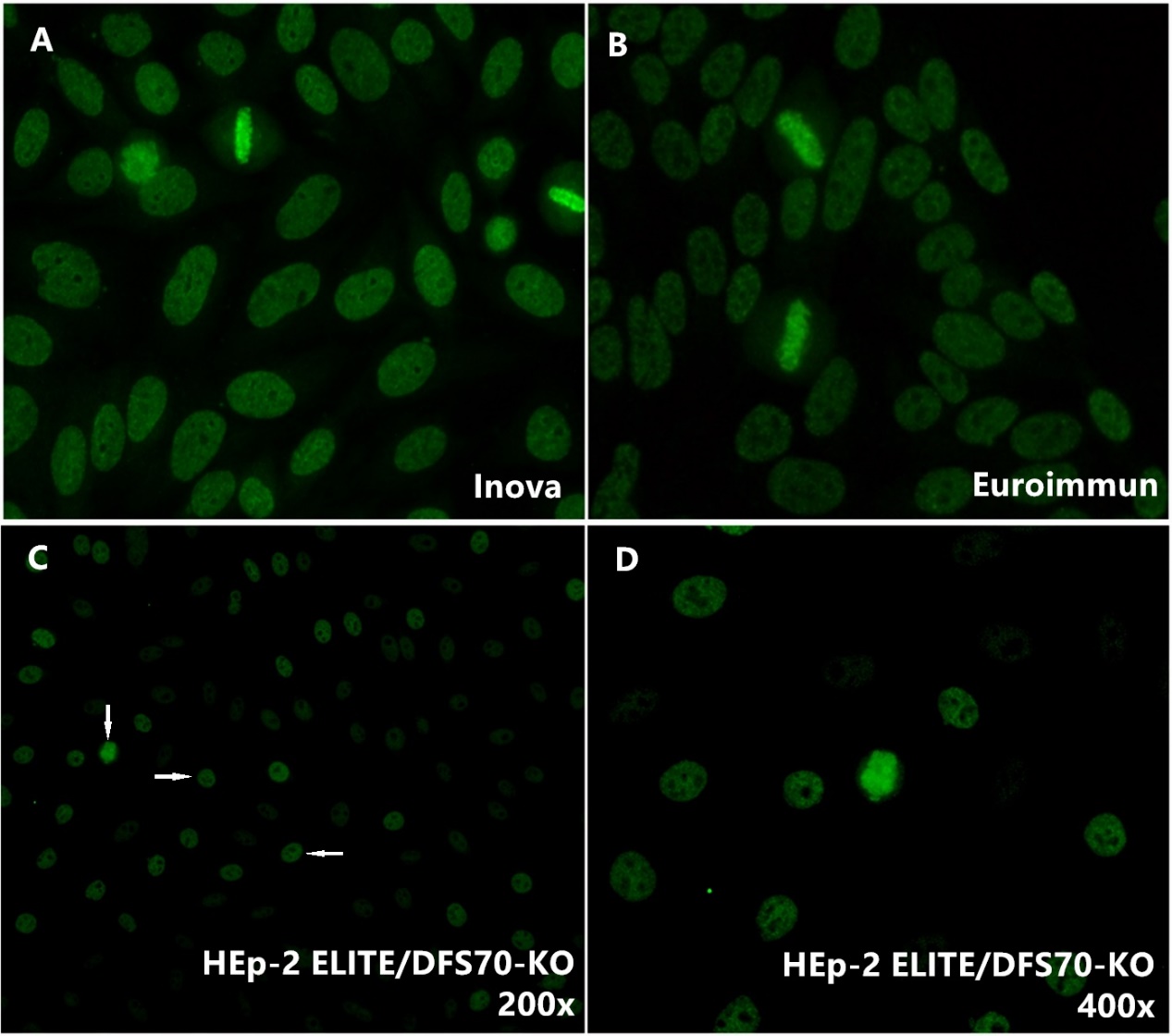


(A) Anti-DFS70 reference material for ICAP pattern AC-2 (CAT#: IS2726) analyzed by Inova slide at a dilution of 1:100. (B) Anti-DFS70 reference material tested by Euroimmun slide at a dilution of 1:80. Both images show the typical DFS pattern with characteristic dense fine speckled staining of interphase nuclei and strong coarse speckled staining of the metaphase plate. (C) Anti-DFS70 reference material performed by HEp-2 ELITE/DFS70-KO substrate (magnification of 200x). Conventional HEp-2 cell nuclei indicated by arrows showed DFS pattern and the DFS70 KO cells were not stained. (D) Anti-DFS70 reference material performed by HEp-2 ELITE/DFS70-KO substrate (magnification of 400x).

**Supplementary Figure 3.** Quantitative correlation between HEp-2 IFA and ELISA.


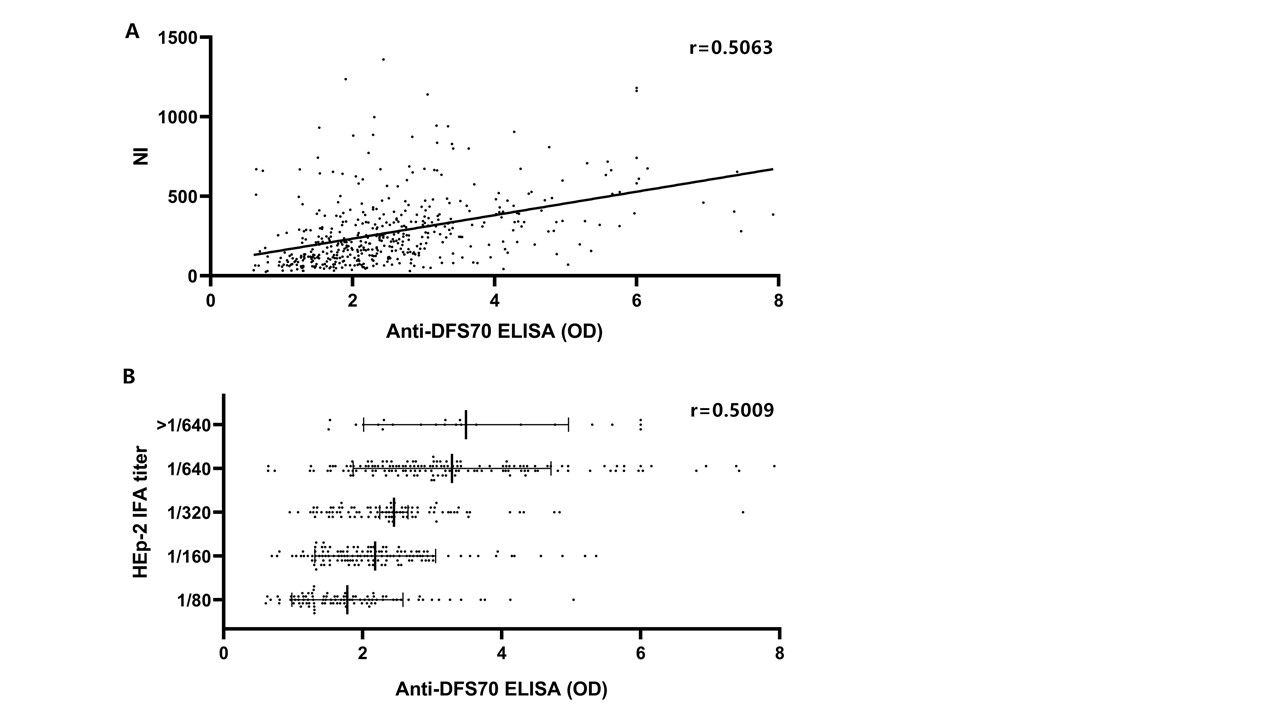


(A) Regression analysis between HEp-2 IFA nuclear intensities (NI) read by NOVA View and anti-DFS70 antibodies concentration by ELISA (O.D. value). (B) Correlation between the HEp-2 IFA titers and the anti-DFS70 antibodies ELISA O.D. value.
